# Molecular Relatedness of Methicillin-Resistant *S. aureus* Isolates from Staff, Environment and Pets at University Veterinary Hospital in Malaysia

**DOI:** 10.1371/journal.pone.0043329

**Published:** 2012-08-24

**Authors:** Erkihun Aklilu, Zunita Zakaria, Latiffah Hassan, Chen Hui Cheng

**Affiliations:** 1 Faculty of Veterinary Medicine Universiti Putra Malaysia, Serdang, Malaysia; 2 Faculty of Veterinary Medicine, Universiti Malaysia Kelantan, Pengkalan Chepa, Kota Bharu, Malaysia; University of Iowa, United States of America

## Abstract

Methicillin-resistant *Staphylococcus aureus* (MRSA) has emerged as a problem in veterinary medicine and is no longer considered as a mere nosocomial pathogen. We studied the occurrence of MRSA in veterinary personnel, cats and dogs and the environmental premises in University Veterinary Hospital (UVH). We found the prevalence of MRSA as follows: UVH 2/28 (7.1%) staff, 8/100 (8%) of the pets [5/50 (10%) of the dogs and 3/50 (6%) of the cats)], and 9/28 (4.5%) of the environmental samples. Antibiotic sensitivity tests (AST) show multi-resistance characteristics of the MRSA and the minimum inhibitory concentration (MIC) values for the isolates ranged from 1.5 µg to >256 µg/ml. Molecular typing by using multi-locus sequence typing (MLST), staphylococcal protein A typing (*spa* typing) and pulsed-field gel electrophoresis (PFGE) was conducted and the results from MLST indicated that an isolate from a veterinary personnel (PG21), typed as ST1241 belonged to the same clonal complex (CC) as the two isolates from two dogs (DG16 and DG20), both being typed as ST59. The PFGE results revealed that the two isolates from two veterinary personnel, PG21 and PG16 belonged to closely related MRSA strains with isolates from dog (DG36) and from environmental surface (EV100) respectively. The fact that PFGE revealed close similarity between isolates from humans, a dog and environmental surfaces indicates the possibility for either of them to be the source of MRSA and the potential routes and risks of spread.

## Introduction

Reports of MRSA in the community beyond the hospital environment suggest the increasing prevalence of MRSA in humans [1]. In the past few years, there have been increasing reports of MRSA in companion animals [2], [3] and veterinary professionals [4], [5], [6] which has made MRSA as a potential emerging problem in veterinary medicine [5] and human hospital environments [7].

Studies have shown that transmission of MRSA can occur from human to animal and vice versa and direct exposure to MRSA-positive animals may lead to transmission to humans [7], [8], [9]. The environmental contamination by MRSA has been implicated as sources of infections in human [10] and veterinary hospitals [11]. MRSA strains have been found to survive for long periods on many different surfaces in the hospital environment and in private homes [12], [13].

Molecular typing methods have been used to track the sources and transmissions of pathogenic bacteria, thereby helping the establishment of national and global epidemiological data of pathogens like MRSA [3], [14], [15], [16], [17], [18]. Among the molecular typing methods, PFGE has been considered as a highly discriminatory method and as the ‘gold standard’ for MRSA outbreak investigations [14]. The advantages of using PFGE for molecular typing are attributed to full typability of isolates, good reproducibility of results within centers and recognizable stability of genomic pattern relatedness over years and high discriminatory power [19]. PFGE has been used as a tool for monitoring and tracking the transmission and spread of MRSA between human and animals [3], [5], [15], [20], [21]. In addition, sequence-based typing methods such as MLST and *spa* typing have been used to study the evolution and epidemiology of MRSA [22], [23].

In Malaysia, MRSA have been reported at 44.1% detection rate in teaching and referral hospital in Kuala Lumpur [24]. However, little is known about the molecular characteristics and MRSA prevalence in the veterinary settings. The current study described the isolation of MRSA in a local veterinary setting and the genetic relatedness of isolates from veterinary personnel, cats and dogs, and environmental surfaces.

## Materials and Methods

### Ethics Statement

Approval for the study was acquired from the university ethics committees for research involving humans and animals (University Committee for Medical Research Ethics and Committee for Ethics for Using Animals in Research). All human subjects involved here were breifed and signed a consent form. The data were also analysed anonymously.

### Sampling

Sampling was conducted in such a way that the staff members are sampled over a one week period, while sampling from pets and environment was done over six months time period. All samplings were done once and there was no replicate sampling. A total of 28 staff, 100 pets (50 cats and 50 dogs) presented to UVH, and 200 environmental surfaces at UVH, Faculty of Veterinary Medicine, Universiti Putra Malaysia (UPM) were sampled between November 2007 and May 2008. The UVH staff members provided informed consent for voluntary participation in the study. Both nasal and oral swabs were collected from each staff member. From pets, nasal and peri-anal swabs were collected by using separate sterile swabs. Environmental sampling was done on selected surfaces of approximately 25 cm^2^ areas at small animal hospital (waiting areas, chairs, reception desk, floors, examination table, markers, water taps, door handles), small animal ward (examination tables, cages), surgical and radiology wards. Sterile swabs were rinsed with sterile normal saline solution to wipe the selected surfaces for sampling. All samples were placed in Amies transport medium (Amies, Italy) and were kept at 4°C until processed.

### Isolation and Phenotypic Characterization

Swab samples were enriched in Tryptone Soya Broth (Oxoid, UK) containing 6.5% NaCl at 37°C for 24 hours prior to culturing [25]. Enriched growths were cultured onto blood agar with 7% horse blood and incubated aerobically at 37°C for 18–24 h. Gram-staining, catalase and coagulase tests were used to identify *S. aureus*. Further confirmations of *S. aureus* isolates were done by culturing on mannitol salt agar (MSA, Oxoid, UK) and by latex agglutination test using Staphytect Plus® (Oxoid, UK). Oxacillin-resistant screening agar base (ORSAB, Oxoid, UK) supplemented with ORSAB selective supplement consisting of 1 mg oxacillin and 25,000 IU polymyxin B incorporated into 500 mL of the agar solution was used for selective growth of MRSA. The plates were aerobically incubated at 37°C for 24–48 h and colony morphologies were used to confirm the growth of presumptive MRSAs.

### Antibiotic Sensitivity Test and MIC Determination

Antibiotic resistance profile for each MRSA isolate was determined by disc diffusion methods according to CLSI standards [34] (2006). Amikacin (AK30), Amoxicillin (AML25), Methicillin (MET10), Oxacillin (OX1), Cefoxitin (FOX30), Streptomycin (S10), Vancomycin (VA30), Minocycline (MH30), Rifampicin (RD15), Doxycycline Hydrochloride (DO30), Amoxycillin-Clauvulanic acid (AMC30), Gentamicin (CN10), Impenem (IPM 10), Tetracycline (TE10), Erythromycin (E15) were the antimicrobials used. Isolates with intermediate and full resistance to vancomycin were further tested by vancomycin Etest.

The Oxacillin MIC for each isolate was determined by using Oxacillin Etest (AB Biodisc, Solana, Sweden) strips according the manufacturer’s recommendations. A pure isolate of MRSA from overnight growth on blood agar was emulsified in normal saline (0.85% NaCl) to achieve a turbidity equivalent of 0.5–1.0 McFarland standard. Isolates with MIC ≥4 µg/mL were considered oxacillin resistant [26].

### Detection of *mec*A, MLST and *spa* Typing

*Staphylococcus aureus* specific gene (*nuc*A) and methicillin-resistance gene (*mec*A) were amplified as described earlier [27]. Two human isolates, three pet isolates and two environmental isolates were typed by MLST and *spa* typing. Multilocus sequence typing was conducted as previously described [18]. The single-locus DNA repeat region of the Staphylococcus protein A gene (*spa*) was sequenced as described previously [28].

### Pulsed-Field Gel Electrophoresis (PFGE)

Pulsed-Field Gel Electrophoresis of *Sma*I (Sigma) digested chromosomal DNA was conducted according to the Harmony protocols [29]. Briefly, bacterial colonies from overnight growth were incorporated into agarose plugs. The PFGE was done by using contour-clamped homogeneous electric field (CHEF) (Bio-Rad, Hercules, California) and was ran in two blocks with a total run time of 23 h; the first block switch time was 5 to 15 s for 10 h, and the second-block switch time was 15 to 60 s for 13 h. The voltage for the run was 6 V/cm or 200 V. The included angle was 120° and the ramping factor was linear. Gels were stained and analysed visually and using Bionumerics® software package Version 3.0 (Applied Math, Sint-Martens, Belgium), using the Dice coefficient and represented by unweighted pair group method using the arithmetic averages (UPGMA) clustering method with 1% band position tolerance and 0.5% optimization settings. A similarity cut-off of 80% [30] and criterion of a difference of ≤6 bands [31] were both used to define a cluster.

## Results

### Prevalence

About 7.1% of the UVH staff (2/28), 8% (8/100) of the pets [5/50 (10%) of the dogs and 3/50 (6%) of the cats)], and 4.5% (9/28) of the environmental samples were found to be MRSA positive based on selective growth on ORSAB.

### Antibiotic Resistance and MIC

All but two environmental isolates were resistant to Oxacillin. The two isolates show intermediate resistance to OX1, however, 4 µg/mL Oxacillin MIC values were recorded for both. The oxacillin MIC values for the isolates ranged from 4 µg/mL to ≥256 µg/mL with an isolate from a cat and two environmental isolates showing the highest MIC values. The two human isolates showed multi-resistance including intermediate resistance against vancomycin. An environmental isolate, EV017 has shown resistance to vancomycin while other two isolates, EV080 and EV100 from the same source were intermediately resistant to the same antibiotic. Moreover, these two isolates had the highest MIC value ≥256 µg/mL (Table 1). However, these three isolates were typed as vancomycin susceptible with MIC values of 0.5 µg/mL for EV080 and EV100 and 1.5 µg/mL for EV017 by Etest.

**Table 1:** Phenotypic and genotypic characteristics of MRSA Isolated from veterinary personnel, pets and environmental surfaces.

Isolate Source and ID			Antibiotic Resistance Profile															Oxacillin MIC (µg/mL)	*nucA*	*mec*A	*spa* type/Repeat Pattern	MLST
			AK30	AML25	MET10	OX1	FOX30	S10	VA30	MH30	RD15	DO30	AMC30	CN10	IPM10	TE10	E15					
**UVH Staff**	Nose	PG16	R	R	R	R	R	R	I	R	R	S	R	R	R	R	R	16	(+)	(+)	t2636 UJGAGJ	ST5
	Nose	PG21	S	S	R	R	S	R	I	R	R	S	S	S	S	R	R	8	(+)	(+)	UJGEGEEJJ	ST1241
**Dogs**	Nose	DG13	R	R	R	R	R	I	S	S	S	S	S	S	S	S	S	12	(+)	(+)	–	–
	Nose	DG16	R	R	R	R	R	R	S	S	S	S	R	S	S	S	R	16	(+)	(+)	t3590 ZAMDMOB	ST59
	Nose	DG20	S	R	R	R	S	S	S	S	S	S	S	S	S	S	S	8	(+)	(+)	t267 UJGFMBBBPB	ST59
	Nose	DG36	R	R	R	R	R	R	S	S	S	S	R	S	S	R	R	16	(+)	(+)	–	–
	Nose	DG49	R	R	R	R	R	R	S	S	S	S	R	S	S	R	R	12	(+)	(+)	–	–
**Cats**	Nose	CT04	R	R	R	R	R	R	S	R	R	R	R	R	S	R	R	≥256	(+)	(+)	t346 UJGBGGJAGJ	ST55
	Nose	CT27	R	R	R	R	R	S	S	S	S	S	S	I	S	S	S	32	(+)	(+)	–	–
	Nose	CT33	I	R	R	R	S	S	S	S	S	S	R	S	S	S	S	32	(+)	(+)	–	–
**Environmental Surfaces**	Waiting area	EV007	R	S	R	R	R	S	I	R	R	R	R	R	R	R	R	24	(+)	(+)	–	–
	Reception desk	EV017	R	R	R	R	R	S	R	R	R	R	R	R	I	R	R	≥256	(+)	(+)	–	–
	SAW table	EV035	R	R	R	R	R	R	I	R	I	R	R	R	I	R	S	12	(+)	(+)	–	–
	SAW cage	EV039	R	R	R	R	S	I	S	R	S	S	R	I	S	R	S	8	(+)	(+)	–	–
	Radiology room	EV041	R	R	R	R	R	R	S	R	R	R	R	R	S	R	S	8	(+)	(+)	–	–
	Surgical ward	EV080	R	R	R	R	R	R	I	S	R	R	R	R	R	R	S	≥256	(+)	(+)	UKGEMBKBGK	ST658
	Surgical ward	EV100	R	R	R	R	R	R	I	R	R	R	R	S	S	R	S	≥256	(+)	(+)	UJGEGE3KELO	ST1156
	SA examinationroom (table)	EV122	S	R	R	I	S	S	S	S	S	S	R	S	S	R	S	4	(+)	(−)	–	–
	SA examinationroom (floor)	EV157	R	R	R	I	S	R	S	S	S	R	R	S	S	S	S	4	(+)	(−)	–	–

SA: Small Animal; SAW: Small Animal Ward; R, Resistant; I, Intermediate Resistance; S, Susceptible.

### Detection of *mec*A, MLST and *spa* Typing

Among the 19 culture positive isolates, 17 (89.5%) were *mec*A-positive, whereas the remaining two isolates from the environment (10.5%) were *mec*A negative. The *mec*A-negative isolates were considered as borderline Oxacillin resistant based on combination of phenotypic features such as selective growth on ORSAB, AST and MIC values. The seven isolates analyzed by MLST were grouped into three clonal complexes (CCs) and two singletons. A human isolate (PG21), typed as ST1241 belonged to the same clonal complex CC59 as the two isolates from dogs (DG16 and DG20), both being typed as ST59. While all the same isolates typed by MLST were assigned to unique *spa* types which shared no similarity in their repeat patterns (Table 1).

### PFGE

**Figure 1:**
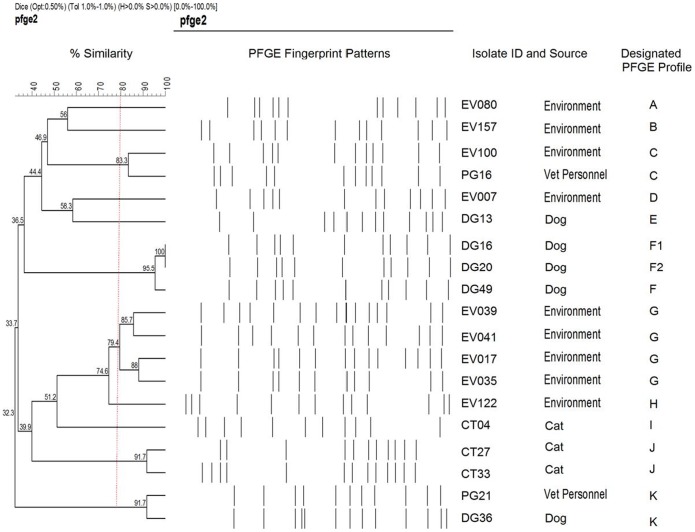
Dendrogram showing PFGE analyzed MRSA isolates from veterinary personnel, cats, dogs, and environmental surfaces at UPM. Data was analyzed using BioNumerics® (Applied Math, Sint-Martens, Belgium) Version 3.0. Dendrogram were derived from the unweighted pair group method using arithmetic averages (UPGMA) and based on Dice coefficient at band optimization of 0.5% and 1% band position tolerance.

Fingerprinting by PFGE grouped the isolates into 11 PFGE profiles. Close similarities were seen in isolates from dogs, cats and environmental surfaces. Three of the five isolates from dogs, two of the three isolates from cats and four of the nine environmental isolates were designated as genetically related based on their PFGE profiles. An isolate from dog (DG36) and a human isolate (PG21) from veterinary personel shared more than 90% similarity in their PFGE profile and hence were grouped as having genetic similarity. Likewise, an isolate from veterinary personnel (PG16) had 83.3% PFGE profile similarity with an isolate from environmental surface (EV100) indicating the close similarity between the two (Fig. 1).

## Discussion

There has been increase in the number of reports of the isolation of MRSA from veterinarians and companion animals [3], [21], [32], [33]. This underscores that MRSA is an emerging problem in veterinary medicine. The current study isolated MRSA from veterinary personnel, pets, and environmental surfaces in small animal hospital. A similar study in the UK found that the epidemic MRSA strain (EMRSA-15) occur in staff, patients and environmental sites in the referral small animal hospital [21]. While concurrent colonization with MRSA has been identified in humans and animals, MRSA can be transmitted between humans and animals many times within a household or veterinary clinic [34].

In a recent study conducted in Japanreported that being in contact with an identified animal MRSA case and being an employee of a veterinary hospital are the two independent factors associated with MRSA carriage [35]. They also suggested that animal patients spread MRSA infection among human individuals in veterinary hospitals. Recent evidence indicates that the environment in veterinary hospitals may be a potential source of MRSA [36]. It has been implicated that a clone of MRSA may have spread from a veterinarian to a dog patient by direct contact or through the medium of contaminated environments [35]. Our findings are supported by another study that reported MRSA at 12% (19/157) of the veterinary hospital environments sampled [36]. According to a recent report [37], humans were found to represent the most important source of MRSA for dogs in both community and veterinary hospital settings. The environment was found to be secondary to humans in terms of importance and other dogs less still [37]. A contaminated environment has also been reported as a potential source of MRSA in human and animal hospitals. It was stated that MRSA contaminated environment can be a source of contamination to the gloves of healthcare workers and hence the pathogen gets transmitted to patients. A study conducted in Canadareported a widespread contamination of the veterinary hospital, which suggested the environment as an important source of MRSA infection [11]. MRSA contamination in veterinary hospitals has been reported in previous studies [21], [37]. Though there is no evidence showing the direct transmission of MRSA from the environment to patients [38], the environment may serve as a source of MRSA exposure for animal health workers.

The PFGE pattern revealed that an isolate from a dog is closely related to an isolate from veterinary personnel in the present study. Likewise, an isolate from environmental surface was found to be closely related to a human isolate from other veterinary personnel. In a study conducted in Germany, an isolate from a cat was reported to be indistinguishable from the PFGE pattern of the human epidemic strain Barnim [39]. Furthermore, two studies conducted in UK [14] and Ireland [20] have respectively demonstrated that MRSA isolates in dogs and cats were indistinguishable or closely related to their attending personnel on PFGE. A similar finding has reported the genetic relatedness between MRSA isolates from human and environmental surfaces in veterinary teaching hospitals in UK [21]. In addition, MLST results in this study showed that a human isolate belonged to the same clonal complex (CC59) with two isolates from dogs, indicating the similar genetic background among the isolates. The fact that human isolates were closely related to isolates from a dog and environmental surfaces indicates the potential for either of the sources to serve as reservoirs for the other. This further implies the possibility of spread of the MRSA clones to an epidemic level, which in turn enables the strains to circulate and prevail in the small animal hospital premises and veterinary personnel. Furthermore, it is possible for veterinary staff to redistribute the strains into the community. A study has documented MRSA transmission between humans and dogs as they found the same MRSA strain in three staff members of small animal and equine hospital and three dogs, all being identical to the predominant human epidemic strain EMRSA-15 [40]. Other studies have also reported the similarity or close similarity of MRSA isolated from humans and companion animals [14], [20], [39], [41].

Since *S. aureus* is resistant to desiccation and can survive longer in the environment [42], [43], it is possible that a contaminated environment can serve as a source of colonization or infection with the bacteria. The environment has been indicated as potential source of MRSA in veterinary hospitals [36], [37]. The significant contribution of environment in maintenance and propagation of MRSA was the possibility in the current study. However, further detailed studies are needed to affirm the importance of environment in MRSA transmission and its potential to serve as a source of infection in veterinary settings for animals and humans as well. In connection with this, the role of veterinary personnel as a possible vehicle for MRSA introduction into animal hospitals needs to be studied further and addressed to develop a sound MRSA control and prevention strategy. Though this study had limitations in ruling out the source of MRSA isolates in any of the sources, we believe that it paves the way for a more comprehensive study to be conducted in Malaysia.
